# Accurate diagnosis of isolated iliac vein thrombosis in third trimester pregnancy with clues on great saphenous vein reflux: a case report and review of literature

**DOI:** 10.1186/s12884-023-05412-z

**Published:** 2023-02-08

**Authors:** Zhanghong Wei, Jingzhi Li, Lijun Liang, Hui Luo

**Affiliations:** 1 Department of Ultrasound, Shenzhen People’s Hospital, The Second Clinical Medical College of Jinan University, The First Affiliated Hospital of Southern University of Science and Technology, 518020 Shenzhen, Guangdong People’s Republic of China; 2 Department of Maternity, Shenzhen People’s Hospital, The Second Clinical Medical College of Jinan University, The First Affiliated Hospital of Southern University of Science and Technology, 518020 Shenzhen, Guangdong People’s Republic of China; 3 Department of Nursing, Shenzhen People’s Hospital, The Second Clinical Medical College of Jinan University, The First Affiliated Hospital of Southern University of Science and Technology, 518020 Shenzhen, Guangdong People’s Republic of China

**Keywords:** Deep venous thrombosis, Iliac vein, Pregnancy, Great saphenous vein reflux, Ultrasound, Case report

## Abstract

**Background:**

Pregnancy is known to be a risk factor for venous thromboembolism (VTE). We report the case of a pregnant patient with difficult to diagnose iliac vein thrombosis, establishing a definite diagnosis by clues of great saphenous vein reflux.

**Case presentation:**

A 37-year-old G1P0 woman at 35 weeks of assisted twin gestation presented with a complaint of persistent left lower limb edema and tenderness. A vascular ultrasound was used to examine the bilateral lower limb. Doppler of left lower extremity revealed continuous great saphenous vein reflux. Right saphenofemoral veins demonstrated venous stasis and no reflux. Unilateral continuous great saphenous vein reflux suggested left iliac veins obstruction or extrinsic compression. Anterograde venography showed a completely occlusive filling defect of the left external iliac vein, which is the definitive diagnosis of acute deep venous thrombosis. The patient underwent a cesarean delivery following inferior vena cava filter (IVCF) placement, and no signs of deep venous thrombosis (DVT) or pulmonary embolism (PE) were reported after delivery.

**Conclusion:**

In pregnant women with suspected deep vein thrombosis, it is imperative to assess the presence of unilateral continuous great saphenous vein reflux.

## Background

Pregnancy is one of the major risk factors in the development of venous thromboembolism (VTE), with pregnant women having a fourfold increased risk of VTE compared with non-pregnant women [1]. Pregnancy-associated VTE is an important cause of maternal morbidity in the United States, accounting for 9.3% of maternal deaths [2]. Isolated iliac vein thrombosis will lead to a series of symptoms such as abdominal pain, back pain, and/or swelling of the entire leg [3]. The diagnosis of suspected DVT in pregnancy is difficult, as pregnancy itself may induce similar symptoms. This case study demonstrates the importance of focusing on the unilateral continuous great saphenous vein reflux of pregnant patients in clinical practice.

## Case presentation

### Chief complaints

A 37-year-old pregnant woman G1P0 at 35 weeks of twin gestation complained of left leg swelling and pain for approximately 1 week. In an external imaging facility, she underwent a lower extremity ultrasound, which revealed varicosis of the left great saphenous vein.

*History of past illness *(Table 1)

*Personal and family history *(Table 1)

**Fig. 1:**
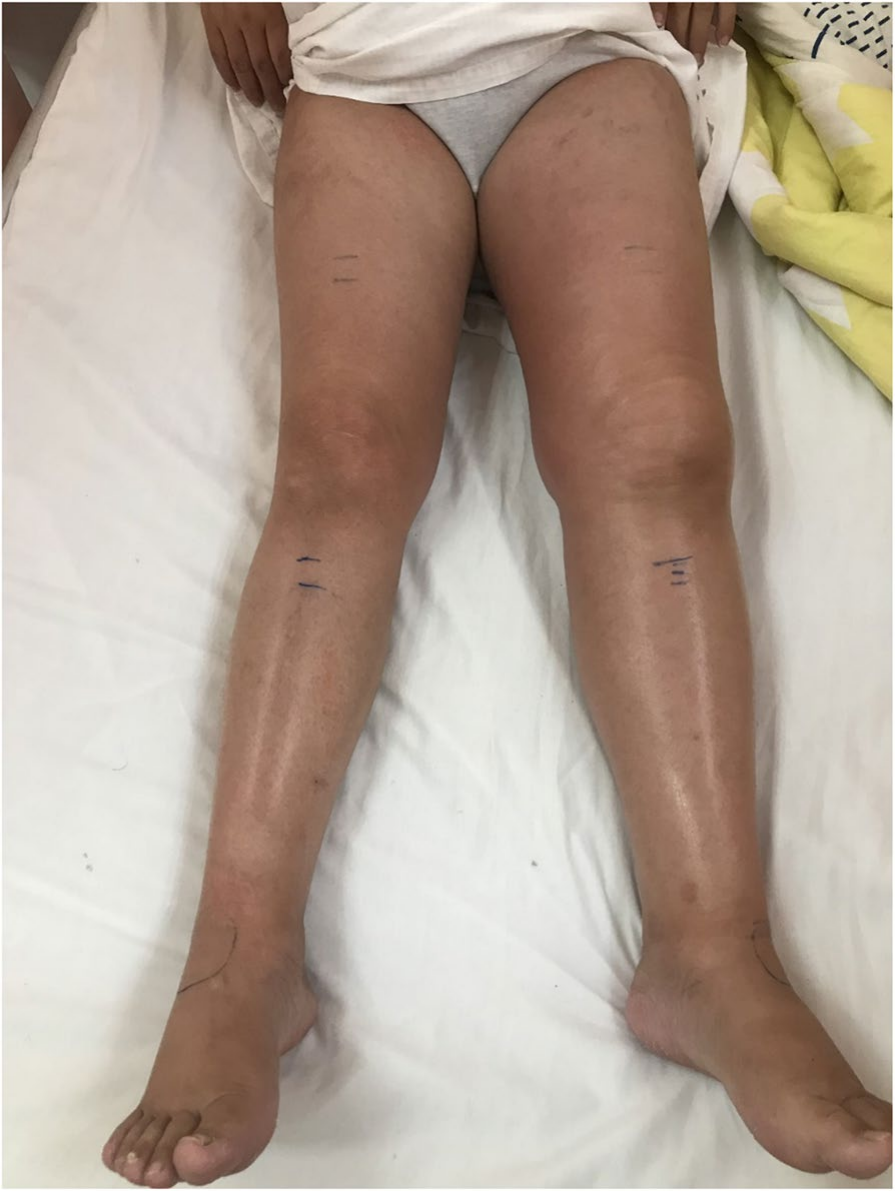
The initial visit of the Patient

*Physical examination *(Table 1, Fig. 1)

### Timeline

**Table 1:** Timeline

Dates	Relevant Past Medical History and Interventions		
	A 37-year-old pregnant woman G1P0 at 35 weeks of assisted twin gestation, neither she nor anyone in her family had a history of thromboembolic disease.		
**Date**	**Summaries from Initial and Follow-up Visits**	**Diagnostic Testing** **(including dates)**	**Interventions**
2021/9/15	left lower extremity swelling and tenderness.	Left lower extremity circumference measurements: left thigh = 49.5 cm, left calf = 38.5 cm, right thigh = 45 cm, right calf = 35 cm. Lower extremity vascular ultrasound was negative. D-dimmer > 10000ng/ml.	Low molecular weight heparin (Clexane, 4000 IU) was injected subcutaneously twice daily.
2020/9/18	Repeated lower extremity vascular ultrasound.	Unilateral continuous saphenofemoral reflux in the left lower extremity. D-dimmer > 10000ng/ml.	Low molecular weight heparin (Clexane, 4000 IU) was injected subcutaneously twice daily.
2020/9/19	Anterograde venography.	Anterograde venography showed a completely occlusive filling defect in the left external iliac vein.	IR-guided IVCF placement. Stopped injecting low molecular weight heparin.
2020/9/23	Caesarean delivery.	D-dimmer > 10000ng/ml.	
2020/9/24		D-dimmer 7620.81ng/ml	Low molecular weight heparin (Clexane, 6000 IU) was injected subcutaneously twice daily.
2020/9/25		D-dimmer 2407.94ng/ml	
2020/10/7	Retrieved IVCF	D-dimmer 1829.2ng/ml	

### Laboratory examinations

The patient had an elevated plasma level of D-dimmer(> 10000ng/ml).

### Imaging examinations

**Fig. 2:**
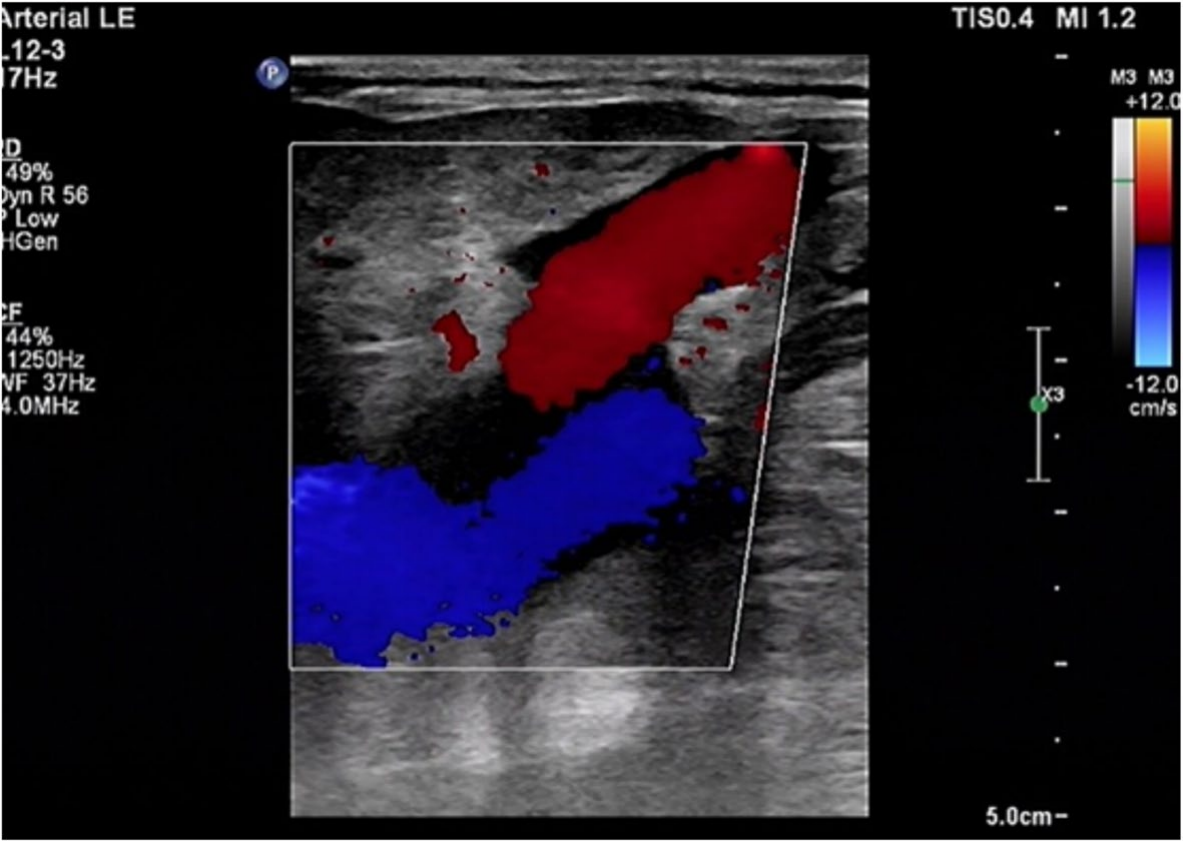
Color Doppler of the left sapheno-femoral junction showed continuous saphenofemoral reflux

**Fig. 3:**
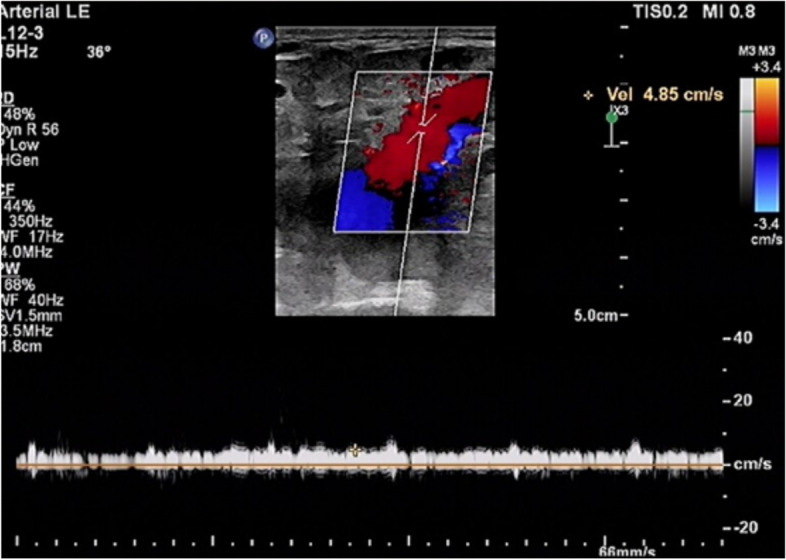
Spectral Doppler of the left great saphenous vein showed continuous reversed venous flow

**Fig. 4:**
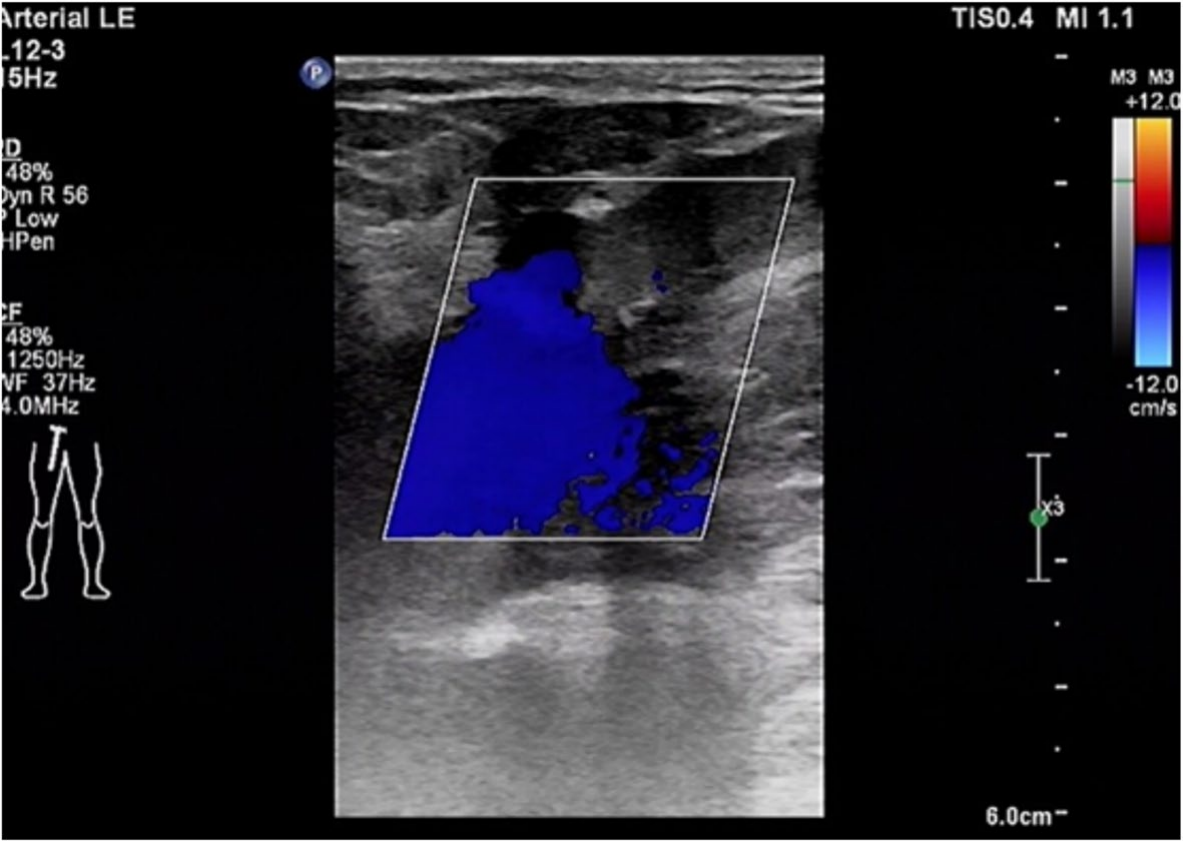
Color Doppler of the right sapheno-femoral junction showed venous stasis without reflux

**Fig. 5:**
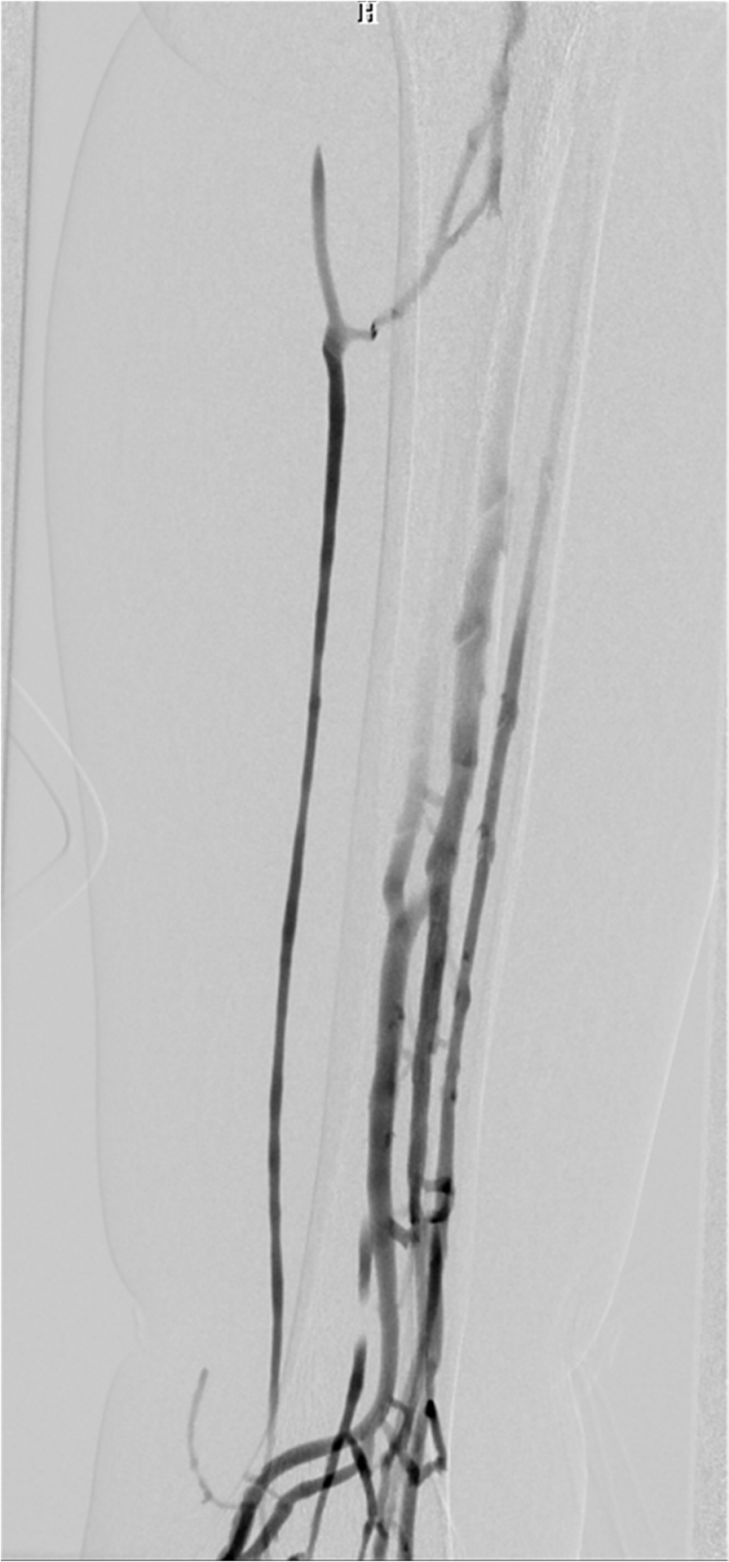
Anterograde venography showed a completely occlusive filling defect in the left external iliac vein

Colour venous doppler ultrasound of the bilateral lower extremity revealed that the deep femoral vein, popliteal vein, proximal segments of crural veins were normal. Imaging revealed unilateral continuous saphenofemoral reflux in the left lower extremity (Fig. 2). The saphenofemoral reflux was monophasic (Fig. 3). Right saphenofemoral veins demonstrated venous stasis and no reflux (Fig. 4). Asymmetry of waveforms suggests left iliac veins obstruction or extrinsic compression. Ultrasound could not visualize bilateral iliac veins because of the huge size of the gravid uterus. In pursuit of a definite diagnosis, anterograde venography was performed on her admission. Anterograde venography showed a completely occlusive filling defect in the left external iliac vein (Fig. 5), which supported the diagnosis made by ultrasound. The patient elected to proceed with IR-guided IVCF placement. The radiation dose was 0.4 mSv. The postoperative course was uneventful. Then the patient underwent a caesarean delivery 2 days later. She delivered two healthy newborns, a male and a female and blood loss was minimal. The boy weight 2290 g, with APGAR scores of 7 at 1st min. The girl weight 2700 g, with APGAR scores of 6. Both newborns were rated 9 points at 5th min in the APGAR scores after administration of oxygen by face mask. The newborns were discharged in good condition on the day of life 6 from the newborn nursery. Since there were no signs of DVT or PE, IVCF was retrieved routinely after 14 days without complication (Table 1).

## Discussion

Pregnancy is a pro-coagulant state [4]. Combined with pregnancy-induced thrombophilia, venous stasis from the gravid uterus quadruples the incidence of DVT during pregnancy [5]. Unfortunately, most of the symptoms that raise suspicion of DVT or PE, such as swelling or pain in the legs, tachycardia, and dyspnea, represent benign physiological changes during pregnancy [6], and most patients with these symptoms will not have DVT or PE.

**Fig. 6:**
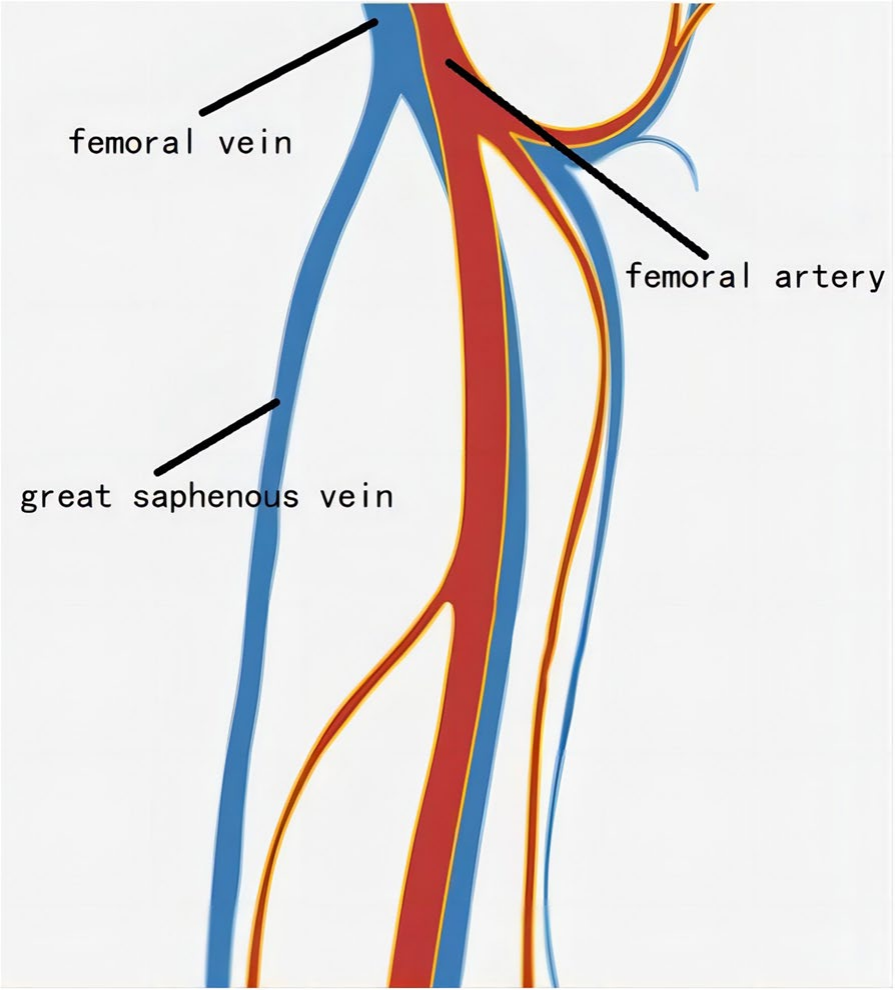
Example of normal great saphenous vein flow

**Fig. 7:**
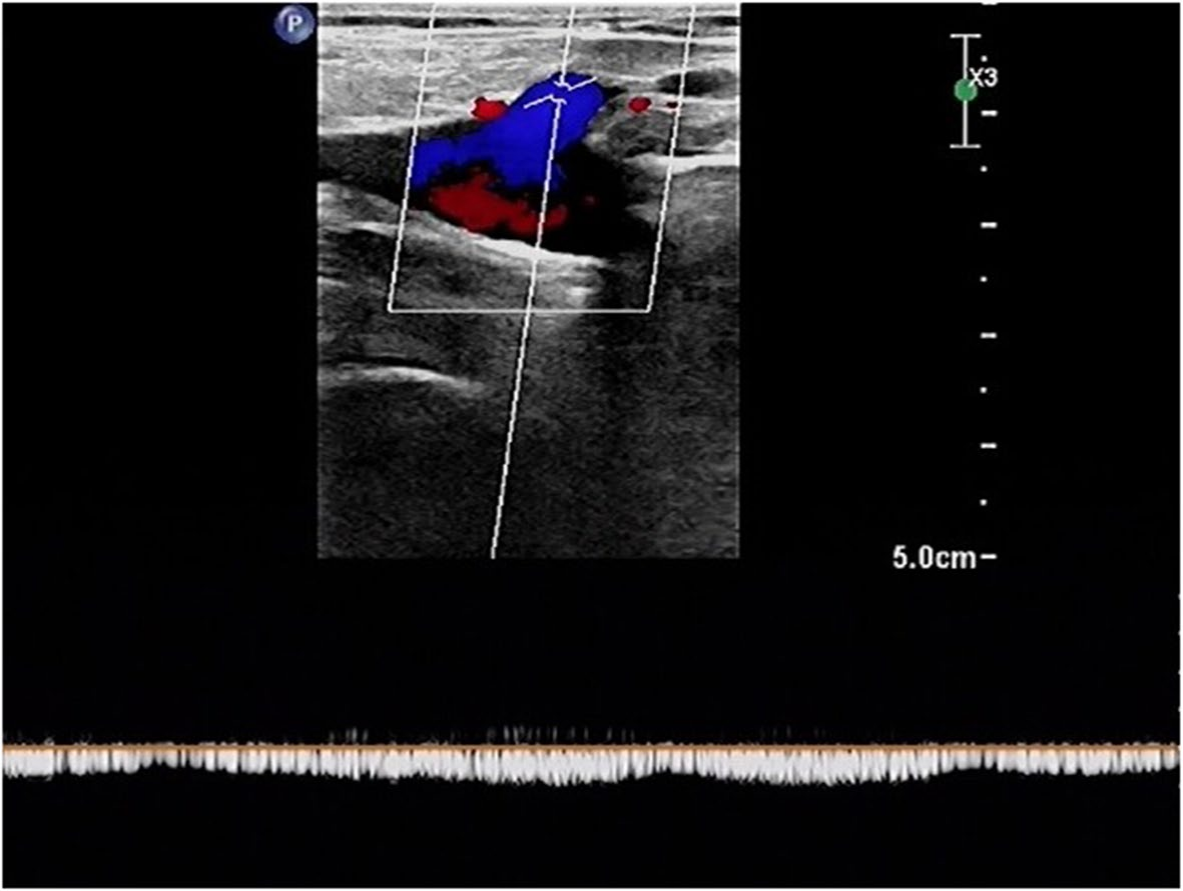
Example of saphenofemoral insufficiency response to valsalva

D-dimer is physiologically elevated during pregnancy, so its utility during pregnancy is limited to ruling out DVT. According to an analysis of a prospective study [7] evaluating the use of US for DVT diagnosis in pregnant patients using whole-blood agglutination D-dimer assays, the sensitivity of the assay was 100% (CI, 77–100%), the specificity was 60% (CI, 52–68%), and the negative predictive value was 100% (CI, 95–100%). Therefore, the diagnostic approach for VTE during pregnancy differs from that in non-pregnant individuals. The recommended initial diagnostic test is compression ultrasonography (CUS) when signs or symptoms suggest new-onset DVT [8]. In a review of published case series [9], in 88% of cases, DVT involved the left leg, and 46% of the described thrombosis was confined to the iliac and/or femoral vein. Notably, 12% of DVTs in pregnant women were isolated pelvic vein thrombosis compared to less than 1% in the non-pregnant population [10]. Left leg and proximal vein involvement in a pregnant patient supports the hypothesis of May–Thurner syndrome (MTS), also known as iliac vein compression syndrome, caused by the right thick-walled common iliac artery compressing the left thin-walled iliofemoral vein. Iliac vein thrombosis can be diagnosed by direct observation of echogenic masses in the cavity or the absence of spontaneous venous flow on Doppler examination (especially with augmentation maneuvers) [11]. Owing to obvious anatomical reasons, a CUS examination cannot be performed over the iliac veins. Because of the deep location, ultrasound imaging is less accurate for iliac vein thrombosis, especially in pregnant patients. In a study using MR imaging in pregnant women with CUS-proven DVT, CUS did not reveal concurrent pelvic vein thrombosis in 11% of patients [12]. When the iliac vein is blocked, blood flow from the lower limbs can bypass the branches of the great saphenous vein and return to the inferior vena cava. Vessels such as superficial circumflex iliac vein, superficial epigastric vein, and superficial external pudenal vein may form collateral circulation. Therefore, reverse blood flow occurs in the saphenous vein. In this case, the reflux in the saphenous vein lacks respiratory phasicity. This reflux is distinct from venous insufficiency with Valsalva response and respiratory phasicity (Figs. 5, 6 and 7). Flow that lacks respiratory phasicity and is not stopped by proximal compression or Valsalva maneuver is called continuous flow [13]. Unilateral continuous great saphenous vein reflux is suggestive of iliac veins obstruction or extrinsic compression. The imaging of reflux in the great saphenous vein may be integrated into the point-of-care clinical ultrasound protocol to predict iliac vein thrombosis during compression ultrasonography of the lower extremities.

The recommended treatment for thromboembolism in pregnant patients consists of weight-based unfractionated heparin or low-molecular weight heparin throughout pregnancy until at least 6 weeks postpartum [2]. The reduction of recurrent deep venous thrombosis or PE in patients who continued to anticoagulants was 64% compared with those taking placebo [14, 15]. In this case, the patient received low molecular weight heparin for anticoagulation, and no bleeding or bruising complications at the time of injection.

IVCF was widely used in the daily clinical practice to prevent PE. An inferior vena cava (IVC) filter can act as a mechanical barrier in the prevention of a lower extremity or pelvic venous thrombosis from embolising to become a potentially lethal pulmonary embolus. The intended dwell time of a temporary IVCF is 7 to 35 days. Complications of IVC filters include trauma at insertion, fracture and/or migration, occlusion by thrombus or endothelialisation, penetration of the IVC and failed retrieval [16].

## Conclusion

Because of the morbidity and mortality associated with pregnancy-related thrombosis, a more accurate DVT diagnosis remains an important focus of future research. We present the case of a patient with suspected DVT in whom to establish a definite diagnosis by great saphenous vein reflux. Anatomical communications between superficial and deep veins should be remembered. In the lower limb, the most important superficial veins are the greater and lesser saphenous veins. Continuous great saphenous vein reflux may be a signal of iliac vein thrombosis.

### Patient’s perspective

During my pregnancy, I had symptoms of my left leg swelling and tenderness. The doctors told me that it might be a thrombosis in my leg, I got several tests; however, the doctors were still unsure about the thrombosis. Vascular ultrasound suggested left iliac veins obstruction or extrinsic compression. Anterograde venography confirmed the diagnosis. Although I was disappointed about the diagnosis, I was glad to hear the doctors had finally a definite diagnosis.

## Data Availability

The datasets used and/or analyzed during the current study are available from the corresponding author on reasonable request.

## Abbreviations

VTE: Venous thromboembolism
IR: Interventional radiology
IVCF: Inferior vena cava filter
DVT: Deep venous thrombosis
PE: Pulmonary embolism
MTS: May–Thurner syndrome
CUS: Compression ultrasound
